# Phytotoxicity risk assessment of diuron residues in sands on wheat, chickpea, and canola

**DOI:** 10.1371/journal.pone.0306865

**Published:** 2024-12-06

**Authors:** Win Win Pyone, Richard W. Bell, Michael T. Rose, Gavan McGrath

**Affiliations:** 1 SoilsWest, Center for Sustainable Farming Systems, Food Futures Institute, Murdoch University, Murdoch, Australia; 2 Cooperative Research Centre for High Performance Soils, Callaghan, Australia; 3 NSW Department of Primary Industries, Wollongbar, Australia; 4 Faculty of Science and Engineering, Southern Cross University, Lismore, Australia; Feroze Gandhi Degree College, INDIA

## Abstract

While diuron residues are being detected more frequently in agricultural soils, there is limited information available regarding their potential phytotoxicity to non-target grain crops. This study aims to determine robust phytotoxicity thresholds for three common, but contrasting, crop species (canola, chickpea, and wheat) exposed to a range of diuron concentrations and to determine how loamy sand soil can change the toxicity thresholds relative to an inert sand. The log-logistic non-linear regression model proved most effective in determining toxicity thresholds by analysing crop responses to diuron. Canola was the most sensitive to diuron in sand followed by wheat and chickpea. Diuron exhibits higher phytotoxicity in sand compared to loamy sand, with ED_50_ values (which is the dose at which diuron causes a 50% decrease in plant growth) of 0.03 mg kg^-1^ and 0.07 mg kg^-1^ for canola shoot biomass inhibition and 0.01 mg kg^-1^ and 0.06 mg kg^-1^ for root dry weight reduction, respectively. The ED_50_ values for wheat shoot biomass (0.11 and 0.24 mg kg^-1^) in sand and loamy sand, respectively, and the ED_50_ values for root growth inhibition are 0.14 mg kg^-1^ in sand and 0.19 mg kg^-1^ in loamy sand. These values were lower than label concentrations and previously estimated average and maximum diuron residue loads (0.17 and 0.29 mg kg^-1^) in Western Australia paddocks. The larger ED_50_ values of diuron in the loamy sand can be attributed to higher soil organic matter and cation exchange capacity that decreased bio-available diuron levels. Average diuron residue loads in Western Australia crop fields exceed the ED_50_ value emphasizes the need for careful planning of crop rotations to avoid crop phytotoxicity from soil-borne diuron residues. Further study is needed to determine the effect of a wider range of soil properties such as pH, clay content, and soil organic matter on the phytotoxicity risk of diuron to rotational crops.

## Introduction

A recent global meta-analysis found that residues of currently used herbicides were present in nearly all monitored agricultural soils, to varying degrees [1]. In many places worldwide, the threat of herbicide-resistant weeds has led to recommendations for increasing the diversity, frequency, and rate of herbicide application to minimise weed survival [2, 3]. However, herbicide carry-over or accumulation in the soil from previous cropping seasons may occur if their frequency of use and persistence exceeds their dissipation rate [4]. Herbicide residues in the soil can be harmful to the growth and development of rotational crops [5–7] reducing yields by up to 35 to 100% when herbicide residues carryover is severe [8]. Additionally, previous researchers found that annual legumes sown as rotational crops or into stubble exhibited impaired root and nodule development as well as shoot necrosis, deformities and stunted growth [9]. In addition to adverse effects on crop production [10–12], there are also concerns about flow-on effects of herbicide residues to natural ecosystems and human health through off-site movement [13–15].

The fate and persistence of herbicides in the soil are influenced by numerous environmental, edaphic, and management factors. Soil properties such as organic carbon content, texture, structure, pH, and microbial activity interact with temperature, rainfall, and management factors to influence herbicide sorption, mobility, and degradation [16–19]. The behaviour of a specific herbicide is further regulated by its physicochemical properties, including water solubility, vapor pressure, ionization constant, and chemical structure [18]. The multitude of relevant factors makes the prediction of herbicide carryover challenging. Sandy soils with low organic matter often have a low capacity to degrade or bind herbicide, which increases herbicide availability to plants compared to heavier-textured, higher organic matter soils [20]. Herbicide carryover therefore often poses a significantly higher risk to the following crops in sandy soil types [20].

Diuron [3-(3,4-dichloro-phenyl)-1,1-dimethylurea] is a widely used herbicide belonging to the class of substituted ureas that can persist in soil for long periods [21]. It is commonly used as a pre-emergence or early post-emergence herbicide to control a variety of grass and broadleaf weeds in both agricultural and non-agricultural lands [22]. Depending on soil properties and, environmental conditions, diuron can persist in soil for over a year due to slow degradation [21, 23, 24]. Sabzevari and Hofman [1] cited several studies on the detection of diuron herbicide residues in the soil [25–29]. Diuron residues were detected in fruit and vegetable farm soils in Spain (0.42–600 μg kg^-1^), in agricultural, grassland, and forest soil in northern France (up to 3.5 μg kg^-1^), and in fallow sugarcane soils in Cuba in the range of 260–2100 μg kg^-1^ [1]. In a soil survey of 84 Australian cropping soils, diuron was one of the most frequently detected herbicide residues, together with glyphosate and its primary metabolite aminomethylphosphonic acid (AMPA), trifluralin and diflufenican [4]. Diuron residues were most frequently detected in soil samples from Western Australian (WA) fields [30] with average and maximum residues loads estimated to be 0.17 and 0.29 kg a.i. ha^-1^, respectively. Because diuron is a broad-spectrum photosystem II inhibitor with long residual activity in soil, diuron has the potential to cause sub-lethal damage to future rotational crops [31]. Due to its efficacy at low concentrations, diuron has been used as a reference herbicide for assessing herbicidal effects on non-target plants [32]. However, there are very few publicly available threshold values reported for assessing the potential toxicity of soil-borne diuron residues to common grain crops [4].

Plant bioassays can evaluate the bio-available levels of herbicide residues in soil that are toxic for plants [33, 34]. Bioassays provide a more complete picture of potential crop damage than chemical analysis as they directly measure the bioavailable rather than total residue level [35]. Dose−response curves derived from herbicide bioassays can be used to estimate toxicity thresholds levels of herbicides for plant species [36–38]. A nonlinear regression model can predict biological response by setting an upper limit at a very low herbicide dose, a lower limit at a very high or lethal dose, and a midpoint at a dose which provides a 50% reduction of plant growth [39]. Effective doses of herbicide that cause 20% or 50% inhibition of the plant growth (ED_20_, ED_50_), can be ascertained through several nonlinear regression models [40, 41]. However, utilization of inappropriate dose−response models may lead to inaccurate estimates of effective dose values.

The organic matter, clay content, cation exchange capacity, and exchangeable cations are positively associated with diuron adsorption to soil [17, 42, 43]. However, the adsorbed diuron can be desorbed and become bioavailable, depending on soil properties [44]. Therefore, determining the concentrations of herbicide residues in different soil types and how this relates to potential damage to subsequent non-target crops is important information in diversified cropping systems. However, the toxicity threshold levels of residual herbicides for key crops are not well known. This knowledge gap is a major limitation to developing management guidelines for avoiding crop phytotoxicity by soil-borne herbicide residues. In this study, diuron was tested due to its importance as a pre-emergence herbicide for crop production in Australia [32], its known persistence and detection in residue surveys, and a lack of publicly available crop toxicity thresholds. The inert sand was selected to determine the distinct phytotoxic effects of known diuron concentrations to contrasting crop species. From that we will derive threshold concentrations of the bioavailable form of diuron on several plant species. This study also evaluated nonlinear regression models to select the best-fitting model for determination of ED_20_ and ED_50_ values. The objectives of this study are to establish phytotoxicity thresholds for wheat, chickpea, and canola, representing diverse crop species, when exposed to soil-borne diuron residues in two soil types: sand and loamy sand soils selected to determine the influence of changes in soil properties on the toxicity thresholds. This information is currently needed to conduct hazard assessments for diuron based on residue levels measured in cropping soils.

## Materials and methods

### Soils, plant species, and herbicide treatments

Dose−response experiments were conducted to assess phytotoxicity of diuron in two soils on three common crop species. Diuron was applied as the commercial product, Diurex WG 900 g kg^-1^ (Nufarm, Australia), which has a recommended rate of 450 g ha^-1^ (405 g a.i. ha^-1^). A loamy sand from a farm field at Meckering, Western Australia was collected from 0–10 cm depth, air dried, and sieved to 2 mm to remove coarse gravel. In addition, a washed coarse sand was purchased (Perth Sand Supplies) and air dried before setting up the glasshouse experiment. The soil properties were determined in the CSBP Soil and Plant Analysis Laboratory by standard methods [45] and results are summarised in Table 1. The loamy sand was packed to a bulk density of 1.4 g cm^-3^ while the sand was packed to a bulk density of 1.6 g cm^-3^.

**Table 1:** Chemical and physical properties of the experimental soils.

Property	Loamy sand	Sand
Organic carbon (%)	0.64	0.13
pH (CaCl_2_)	6.7	8.9
EC (dS m^-1^)	0.041	0.031
PBI	14.9	1.6
Effective CEC (cmol kg^-1^)	1.8	0.2
Clay (%)	0.9	0.6
Coarse sand (%)	87.8	51.8
Fine sand (%)	8.3	46.2
Silt (%)	2.9	1.4

*Note*. EC, Electrical Conductivity; PBI, Phosphorus Buffering Index; CEC, Cation Exchange Capacity

Canola (*Brassica napus* L. cv. ATR Bonito TT), chickpea (*Cicer arietinum* L. cv. PBA Striker), and wheat (*Triticum aestivum* L. cv. Scepter) were tested as common winter crops in Australia. High germination percentages of canola (99%), chickpea (100%) and wheat (100%) seeds were confirmed before the experiment.

### Experimental design and management

To examine toxicity thresholds that disturb crop growth (either root or shoot), a 28-day dose−response experiment was conducted in the glasshouse at the Murdoch University Campus, Perth, Australia. The controlled environment in the glasshouse maintained an average daytime air temperature of 19°C and 36% relative humidity. Eight concentrations of diuron herbicide were applied at rates equivalent to 0, 1/9, 1/6, 1/3, 1, 3, 6, and 9 times the label rate (Table 2). This range of herbicide doses was selected to reproduce the range of herbicide residues encountered under field conditions, to determine crop response to doses, and to assess toxicity threshold levels. The actual application rate of diuron herbicide (mg kg^-1^ soil) for each treatment was calculated based on the field application rate (g ha^-1^). One kg of the commercial product of diuron contained 900 g of active ingredient. To calculate the active ingredient application in g ha^-1^, we estimated the actual amount of herbicide needed (a.i., mg kg^-1^) for each soil type based on its bulk density. The applied rate in units of mass per hectare was converted to concentration considering it to the fully mixed across the soil depth of the pots. Before each experiment, diuron stock solutions were prepared and stored in a refrigerator at 4°C. The amount of diuron (mg kg^-1^ soil) required for each pot was added as a volume of stock solution and diluted with sufficient water to bring the soil to a moisture content of 80% field capacity. Diuron was applied by spraying appropriately diluted herbicide solutions to each soil while rotating in a cement mixer. After mixing with an individual dose of herbicide solution, the mixer was thoroughly cleaned to avoid contaminating the next treatment. The mixing with individual doses of herbicide solution proceeded from the lowest to the highest concentration to avoid contamination of the following treatment. Before seeding, the soils were then incubated for 24 hours in sealed bags in the glasshouse.

**Table 2:** Diuron herbicide rates as the commercial product (Nufarm- Diurex WG 900 g kg^-1^) (g ha^-1^) and their equivalent rates of active ingredient (a.i., mg kg^-1^ soil) applied to loamy sand (bulk density-1.4 g cm^-3^) and washed sand (bulk density-1.6 g cm^-3^).

Relative Label Rate	Product Rate (g ha^-1^)	Loamy Sand a.i. concentration (mg kg^-1^)	Sand a.i. concentration (mg kg^-1^)
0	0	0	0
1/9	50	0.03	0.03
1/6	75	0.05	0.04
1/3	150	0.10	0.08
1	450	0.29	0.25
3	1350	0.87	0.75
6	2700	1.74	1.50
9	4050	2.61	2.25

A randomized complete block design was used with 3 replicates of each treatment combination (i.e., 3 crops x 2 soil types x 8 doses x 3 replications). Plastic pots (0.676 L); dimensions of 16 cm (H) x 6.5 cm x 6.5 cm (W) were each filled with 0.85 kg of soil. Drainage holes at the bottom of the pots were sealed by plastic bags lining inside of the pot. Four seeds were sown in each pot and then covered by a plastic sheet to prevent drying during germination. Plants were thinned to two per pot at 7 days after emergence. During the experiment, each pot was weighed and watered on a daily basis to return water content to 80% of field capacity. The plants were also watered weekly with a mixture of complete nutrient solutions to ensure plant growth was not nutrient limited (S1 Table).

At 28 days after sowing, the plants were harvested and gently washed to remove soil from the roots. All intact plants were patted dry on paper towels after washing. The separated fresh roots and shoots were weighed, and maximum shoot lengths were manually measured with a ruler. Root length was measured with a digital image analysing system (WinRHIZO 2007d, Regent Instrument, Quebec, Canada). Root and shoot dry weight data were collected after materials were dried in an oven at 65°C for 48 hours to a constant weight.

### Data analysis

A three-way factorial analysis of variance (ANOVA) in open-source statistical software R [46] was used to analyse variation of growth inhibition for all tested species in different soil types under the 8 herbicide application doses. After performing the factorial data analysis, a two-way analysis of variance (ANOVA) was used to observe the interaction effect of soil types and crops on the variation of growth inhibition at the label rate. The mean values of growth reduction were compared by using Tukey’s HSD test and P-values were determined to assess the differences between the combination of soil types and crops.

The percentages of shoot and root length and dry biomass inhibition were calculated relative to the untreated control for each crop in each soil type by using the following equation [35],

$$Inhibition(\%)=(1–L_{t}/L_{0})\;x\;100\%$$ (Eq 1)

where L_t_ is the shoot or root length and dry biomass measured in the herbicide-treated soil and L_0_ is the shoot or root length and dry biomass in the untreated soil.

The dose−response curves and ED_20_ and ED_50_ values for canola, chickpea, and wheat were determined by fitting dry weight and length data of shoots and roots against diuron application rates by using the ‘drc’ package [47] in the R statistical software environment. Following the recommendation of Knezevic, Jens [48], the actual biomass and length data were used to calculate ED values for each species and soil types from dose−response curves. For the model selection, Akaike’s information criterion (AIC) was used to identify the best fitting model for this study. Posada and Buckley [49], Sakamoto and Kitagawa [50] suggested that models with smaller (AIC) values demonstrated the superior fits of the models. A lack-of-fit test was also implemented to check the fitting results against the most general model ANOVA in ‘drc’ package [47, 51].

The ED_50_ values (the dosage resulting in a 50% reduction in plant biomass or length) were predicted using various nonlinear regression models [38]. Regression lines were calculated by using four-parameter log-logistic model (LL.4), three-parameter log-logistic model (LL.3), four-parameter Weibull type 1 (W1.4), and Weibull type 2 (W2.4) models to identify the best fitting model for this study. The LL.4 model was selected as the best fit model for the prediction of ED_20_ and ED_50_ values of canola and the ED values of chickpea and wheat were estimated by using the LL.4 function with the biomass and length value set to zero, for representing no further shoot and root emergence at the maximum toxicity level. The dose−response curves were produced by using the ‘drc’ (dose−response curves) package in R [47] and data were plotted using the ‘ggplot2’ package (Wickham, 2016). Equations for all these models are presented [52] (S2 Table).

## Results

### Responses of different species to herbicide doses

In both soil types, plant fresh and dry weights had similar responses to increasing diuron concentrations. Hence the following results focus on dry biomass rather than fresh biomass.

Three-way analysis of variance revealed that the interaction effect of all combinations (S x C), (S x D), (C x D), and (S x C x D) were significant for all evaluated plant growth responses (Table 3).

**Table 3:** Analysis of variance results (mean sum of squares and significance) for the effect of soil types, crop species, and diuron doses on plant and their interactions on crop growth responses.

Source	DF	SDWI	RDWI	SLI	RLI
Block	2	70ns	158ns	25ns	174ns
Soil (S)	1	214ns	2676***	1681***	1272***
Crop (C)	2	11575***	7685***	27213***	9741***
Doses (D)	7	19326***	19292***	11030***	16933***
S x C	2	1104***	384*	907***	373**
S x D	7	1113***	1477***	740***	653***
C x D	14	424***	337***	1650***	508***
S x C x D	14	257***	606***	143***	259***

Note-SDWI-Shoot dry weight inhibition, RDWI-Root dry weight inhibition, SLI-Shoot length inhibition, RLI-Root length inhibition. For each parameter, means with the same letters are not significantly different (P < 0.05, Tukey’s HSD test).

*, **, ***, ns are significant at 0.05%, 0.01%, 0.001% and non-significance, respectively.

Canola was the most sensitive crop in both soil types, followed by wheat and then chickpea. At the label rates, canola shoot and root were reduced by 100% relative to the control in the sand and loamy sand (Table 4). By contrast, wheat shoot dry weight reductions in sand and loamy sand were 63% and 41%, whilst chickpea shoot dry weight reductions were 49% and 61%, respectively. There were similar trends with root dry weight, with the exception that chickpea root dry weight inhibition (77%) was much higher than shoot dry weight inhibition (49%) at label rate in the sand (Table 4).

**Table 4:** Plant biomass and growth inhibition (%) relative to control at the recommended application rate of diuron. Value are means of three replicates ± SE (n = 3).

Crop species	Inhibition (%) in loamy sand				Inhibition (%) in sand			
	SDWI	RDWI	SLI	RLI	SDWI	RDWI	SLI	RLI
Canola	100±0^a^	100±0^a^	100±0^a^	100±0^a^	100±0^a^	100±0^a^	100±0^a^	100±0^a^
Chickpea	61±2.4^b^	71±3.2^b^	16±2.7^c^	54±6^b^	49±1.2^c^	77±0.6^b^	23±2^c^	53±3.4^b^
Wheat	41±3.1^c^	64±0.9^b^	36±2^b^	64±4^b^	63±2.3^b^	54±3.1^c^	40±2.6^b^	53±3.4^b^

SDWI-Shoot dry weight inhibition, RDWI-Root dry weight inhibition, SLI-Shoot length inhibition, RLI-Root length inhibition. For each parameter, means with the same letters are not significantly different (P < 0.05, Tukey’s HSD test).

Shoot and root length were not as sensitive indicators as dry weights. At the label rates in both soils, growth inhibition for chickpea and wheat were in the range of 16–40% for shoot lengths and around 50–60% for root lengths. In contrast, shoot and root length reduction for canola (100%) was significantly greater than for chickpea and wheat in both soil types. Diuron effects were similar in sand and loamy sand for canola shoot and root length response at the label rate, while chickpea root response was significantly higher than shoot length inhibition. Wheat shoot and root length responses were not significantly different (Table 4).

At rates higher than label recommendations, diuron further depressed shoot and root dry biomass and shoot and root length of chickpea and wheat species as expected (S3 Table). Interestingly, at the lower diuron application rates (0.03 mg kg^-1^), wheat shoot and root biomass response presented increased growth relative to the control treatment in the loamy sand. Canola also had higher root biomass and length in the loamy sand at a diuron rate of 0.03 mg kg^-1^, but this was not significantly different from the control treatment (S3 Table).

### Model comparison for dose–response curve fitting

**Fig 1:**
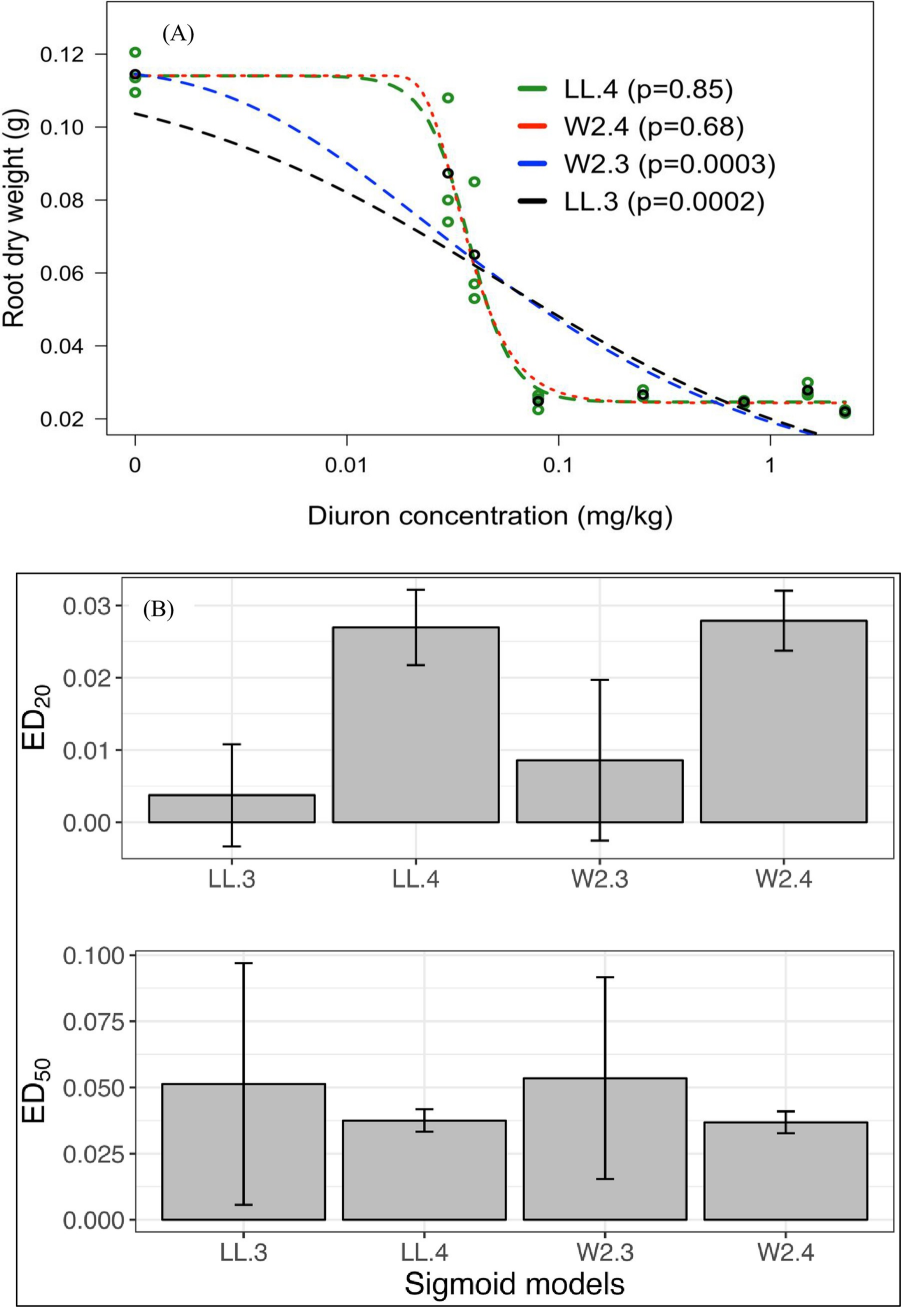
**A. Diuron dose–response curves in sand for root biomass inhibition of chickpea.** Regression lines were calculated by using four-parameter log-logistic model (LL.4), three and four-parameter Weibull type 2 (W2.3 and W2.4) models, and three-parameter log-logistic model (LL.3). P values larger than 0.05 represented better fitting of the models by lack-of-fit test. **B. Comparison of sigmoid models for estimation of diuron effective dose values (ED**_**20**_ **and ED**_**50**_**).** Bars on each column represent standard error.

An example of the model selection result with AIC values for chickpea root biomass inhibition associated with application of diuron herbicide doses and lack-of-fit test results for comparison of ANOVA and DRC models are shown in Fig 1 and S4–S6 Tables.

According to model comparison and lack-of-fit result, LL.4 (p = 0.85) was the best-fitting model, similar to W2.4 (p = 0.68), while W2.3 (p = 0.0003) and LL.3 (p = 0.0002) were inadequate (Fig 1A). Moreover, the ED values in Fig 1B did not differ much among the two sigmoid curves, LL.4 and W2.4 with smaller standard error values, while W2.3 and LL.3 had significantly different values from other models in the case of ED_20_ and ED_50_ with higher standard error values. A similar process was used to identify the best fitting model for each crop-soil combination. These are listed in supplementary (S6 Table).

### Effect of soil properties on diuron phytotoxicity based on dose–response curves

**Fig 2:**
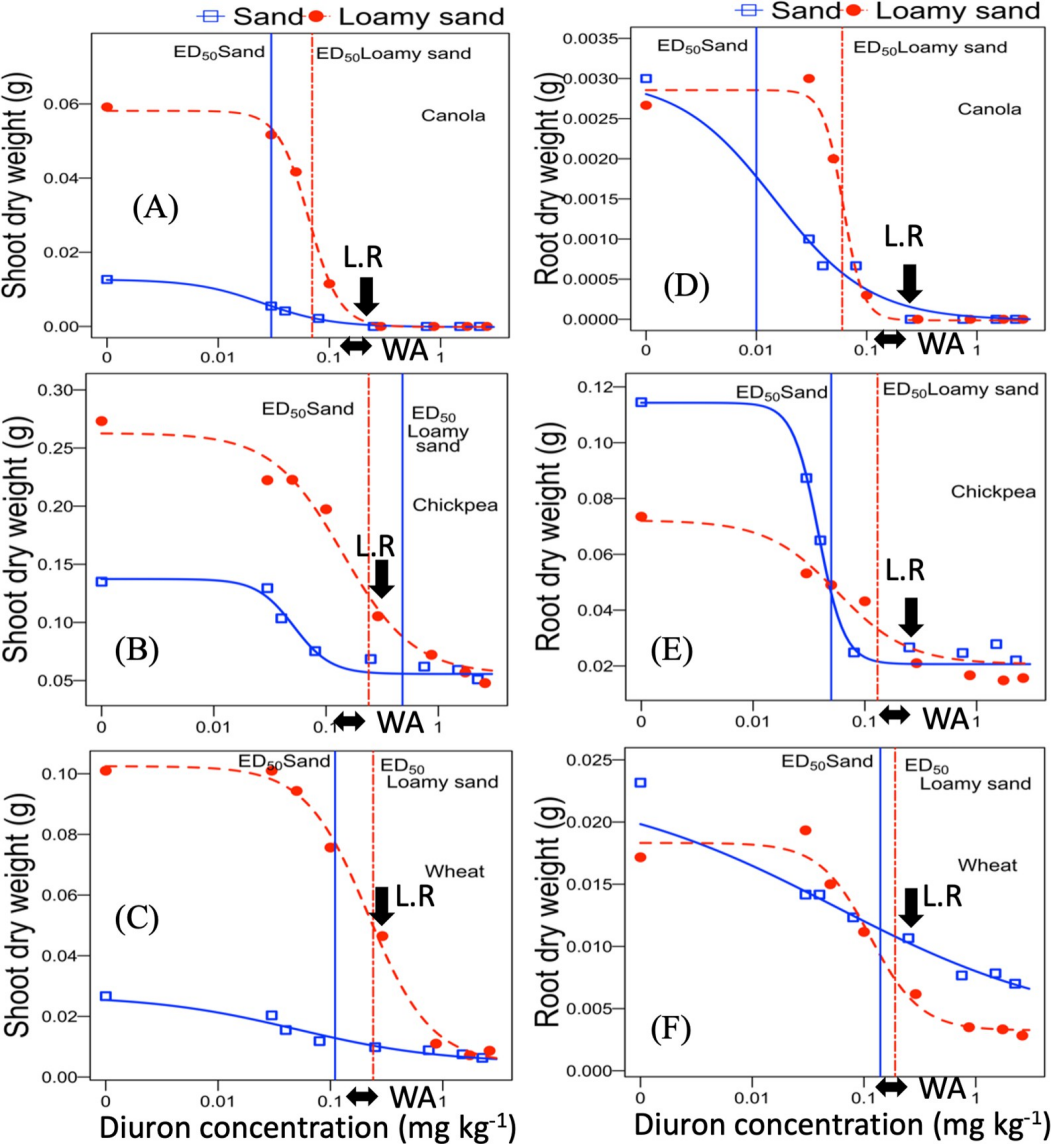
Diuron dose–response curves in sand and loamy sand determined after 28 days for shoot and root dry biomass bioassay for: (A, D) canola, (B, E) chickpea, and (C, F) wheat. Regression lines were calculated by using four-parameter log logistic model and the parameter values are shown in Table 5.—Diuron residue level estimated in Western Australia paddocks [30]. L.R—Label rate.

**Fig 3:**
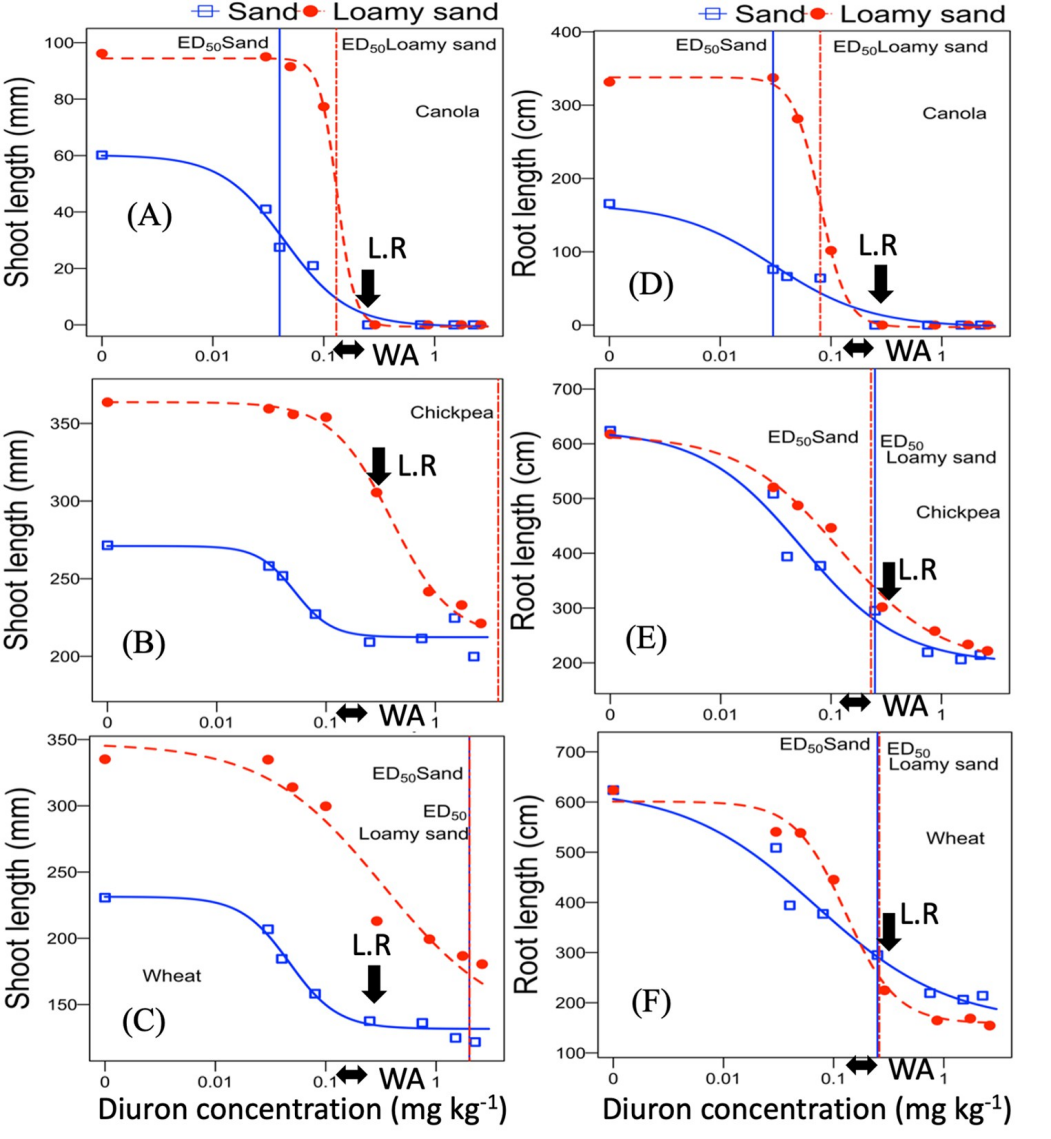
Diuron dose–response curves in sand and loamy sand determined after 28 days for shoot and root length bioassay for: (A, D) canola, (B, E) chickpea, and (C, F) wheat. Regression lines were calculated by using four-parameter log logistic model and the parameter values are shown in Table 5.—Diuron residue level estimated in Western Australia paddocks [30]. L.R—Label rate.

All plant growth parameters decreased with the increasing diuron concentration above threshold values. The ED_20_ and ED_50_ values for diuron inhibition of the shoot and root dry weight and length of canola were estimated by using four-parameter log-logistic model as crops growth were completely suppressed at the label dose. However, chickpea and wheat responses to diuron doses were estimated by three-parameter log-logistic model (LL.4 function with the crops shoot and root parameters set to zero at the maximum toxicity level) as these two species were not completely suppressed until the highest tested application rate in both soil (Table 4; Figs 2 and 3).

It was observed that ED values of chickpea were estimated with a high standard error due to a less severe effect on shoot growth until maximum tested concentration, despite obvious yellowing of shoots and branching declines from the recommended dose.

The ED_20_ values ranged from 0.002 (Wheat-RDW) to 0.62 mg a.i. kg^-1^ (Chickpea-SL) and 0.01 (Chickpea-RDW) to 0.39 mg a.i. kg^-1^ (Chickpea-SL) for sand and loamy sand, respectively. As the estimation of diuron ED_50_ values for chickpea shoot length response were over the maximum tested rates, we excluded this ED value from effective dose comparisons for crop species. The estimated ED_50_ values for sand and loamy sand ranged from 0.01 (Canola-RDW) to 2.0 mg a.i. kg^-1^ (Wheat-SL) and 0.06 (Canola-RDW) to 2.0 mg a.i. kg^-1^ (Wheat-SL) (Table 5). Overall, ED_50_ values of all tested species varied from 34-fold to 200-fold in loamy sand and sand, respectively, showing a wide sensitivity range among the tested bioassay species. The loamy sand had larger ED_50_ values for all wheat and canola response measures indicating lower herbicide phytotoxicity at similar application rates. Although the ED_50_ value for chickpea root dry weight was 2.6-fold higher in loamy sand compared to sand, the effective dose of diuron required to reduce 50% of other growth parameters were lower in loamy sand than in sand (Table 5).

**Table 5:** Herbicide doses corresponding to 20 and 50% plant growth reduction of canola, chickpea, and wheat.

	Loamy sand		Sand	
	ED_20_ ± SE	ED_50_ ± SE	ED_20_ ± SE	ED_50_ ± SE
	Shoot dry weight			
Canola	0.04 ± 0.007	0.07 ± 0.007	0.01 ± 0.002	0.03 ± 0.002
Chickpea	0.04 ± 0.01	0.24 ± 0.05	0.02 ± 0.02	0.48 ± 0.25
Wheat	0.09 ± 0.01	0.24 ± 0.02	0.004 ± 0.02	0.11 ± 0.15
	Root dry weight			
Canola	0.05 ± 0.004	0.06 ± 0.006	0.004 ± 0.003	0.01 ± 0.006
Chickpea	0.01 ± 0.006	0.13 ± 0.04	0.004 ± 0.001	0.05 ± 0.02
Wheat	0.05 ± 0.02	0.19 ± 0.05	0.002 ± 0.001	0.14 ± 0.03
	Shoot length			
Canola	0.1 ± 0.002	0.13 ± 0.009	0.02 ± 0.002	0.04 ± 0.003
Chickpea	0.39 ± 0.1	3.76 ± 0.6	0.62 ± 0.68	16.3 ± 7.8
Wheat	0.13 ± 0.05	2.01 ± 0.44	0.03 ± 0.02	2.0 ± 0.85
	Root length			
Canola	0.05 ± 0.007	0.08 ± 0.006	0.008 ± 0.003	0.03 ± 0.005
Chickpea	0.06 ± 0.02	0.23 ± 0.1	0.01 ± 0.01	0.25 ± 0.09
Wheat	0.04 ± 0.01	0.26 ± 0.05	0.01 ± 0.007	0.25 ± 0.09

Note- Effective doses ED_20_ and ED_50_ (mg kg^-1^) for loamy sand and sand are means ± SE (n = 3).

The order of tested species sensitivity to diuron herbicide, based on effective dose (ED_20_ and ED_50_) values was canola > wheat > chickpea. The diuron concentrations corresponding to 50% inhibition of chickpea shoot length was higher than the highest tested rates in sand and loamy sand (Fig 3B). Wheat shoot length 50% inhibition in the sand and loamy sand was observed around the maximum application rate (2.25 and 2.61 mg a.i. kg^-1^) and it was similar in both soil types (Fig 3C).

Chickpea had the highest ED values for shoot biomass and shoot length reduction, but the herbicide concentration that caused a 50% reduction in root biomass and root length in wheat was higher than chickpea (Table 5).

The ED_50_ values of both soil types for all tested species were lower than the label recommended dose and the diuron residue loads estimated for Western Australia paddocks through soil survey (Figs 2 and 3) except for shoot biomass inhibition of chickpea in sand and shoot length reduction of chickpea and wheat in both soil types.

## Discussion

This study used diuron as a test case and a glasshouse dose−response approach to determine its toxicity in inert sand and in a loamy sand on three contrasting crop species. Different models were tested to evaluate plant growth responses to diuron and to determine which one provided accurate estimations of threshold values for toxicity. In the following discussion, we first examine the most reliable dose−response curve fitting approach to derive reliable ED_20_ and ED_50_ values. Then the variations in ED values of diuron with crop species and soil types are discussed. Finally, the implications of these findings are examined for a more systematic assessment of herbicide toxicity thresholds in soils for major crop species.

### Model comparison for dose–response curve fitting

The log-logistic model was used to describe the dose−response curves following the recommendation of Seefeldt, Jensen [38]. In this study, both of the four and three-parameter log-logistic models (LL.4 and LL.3) mostly produced the best fit for all tested species in both soil types. However, as showed in the example model selection, chickpea root biomass response in sand was fitted by LL.4 and four-parameter Weibull type 2 (W2.4) models (AIC value <10 difference) (S4 Table). Based on studies by, Posada and Buckley [49] and Sakamoto and Kitagawa [50], from the model selection method, AIC value differences with three-parameter log-logistic models (LL.3) and three-parameter Weibull type 2 (W2.3) are over 10, suggesting that the LL.3 and W2.3 models are not suitable for estimation of ED values for chickpea root biomass response to diuron doses. The dose−response curves plotted for all models (Fig 1A) clearly show that LL.4 and W2.4 models are consistent in their prediction of ED_20_ and ED_50_ values for chickpea root biomass.

Additionally, a lack-of-fit test for LL.4 and W2.4 models resulted in (p>0.05) while for the LL.3 and W2.3 models the p-value for lack-of-fit was significant (p<0.05). Hence the present study used the four-parameter log-logistic model for estimation of effective doses of when complete inhibition was not observed, such as for chickpea root biomass. When complete inhibition was reached at higher doses, the LL.3 model was sufficient to estimate ED values.

### Responses of different species on herbicide doses

All species tested in this study showed 40–50% shoot and root biomass growth reduction at rates lower than the recommended application rate in sand and loamy sand. Diuron inhibits photosynthetic reactions of plants through competition with plastoquinone binding at the D1 protein site in photosystem II, retarding the formation of the reduced form of nicotine adenine dinucleotide phosphoric acid (NADPH) and adenosine triphosphate (ATP) [53]. This reaction inhibits CO_2_ fixation by disturbing the electron transport chain and the resulting release of reactive oxygen species (ROS) can cause oxidative damage within plant cells and lead to retarded shoot growth [54]. However, based on the comparison between ED_20_ and ED_50_ values from dose−response analysis, this study found that roots were more sensitive to diuron. Possible reasons for this are discussed below.

The growth of canola was severely inhibited by much lower doses of diuron in both soil types compared to wheat and chickpea. Canola dry biomass and length (Table 4) indicated 100% inhibition in both soil types at the recommended rates for wheat in WA at 405 g a.i. ha^-1^ (0.25 mg kg^-1^ for sand and 0.29 mg kg^-1^ for loamy sand). Herbicide residues from prolonged soil activity could damage vulnerable non-target rotational crops, such as canola, as well as legumes cultivated in subsequent growing season [55, 56]. In previous work, a related Brassica species (turnip; *Brassica rapa*) was the best bioassay species for detecting diuron residues in soil, because it had a low ED_50_ (biomass reduction) of 0.25 mg kg^-1^ in a sandy soil from Wongan Hills, WA [57]. However, the present finding is in contrast with the field studies of Moore and Paul Matson [58], who examined the tolerance of canola (non-triazine tolerant variety) to exposure to individual and multiple herbicides. They suggested that canola was tolerant to pre-planting application of diuron up to 1000 g a.i. ha^-1^ and canola yield even increased at lower rates (250–350 g ha^-1^). The difference between studies could be due to differences in canola varieties, experimental conditions between field and glasshouse studies as well as soil types.

Of the studied species, chickpea was the least responsive to diuron with shoot biomass growth reduced by around 50 and 60% for sand and loamy sand, respectively, at label rates of 0.25 and 0.29 mg kg^-1^. This is in agreement with Vasilakoglou, Vlachostergios [59], who found that the pre-emergence application of diuron at 1000 g ha^-1^ had no phytotoxic effect on chickpea in one season, but observed 20 to 40% injury (based on visual inspection of shoots) at 4 weeks after sowing in the following season. The present research also revealed that root length and dry matter reduction of chickpea by diuron (50–70%) was greater than shoot response at label rate. This may indicate that inhibition of photosynthesis in chickpea by diuron restricted assimilate supply to roots. In addition, it might be due to root or root hair damage since diuron is also absorbed by roots and translocated through the xylem [8]. Phytotoxic effects of diuron herbicides on chickpea root systems have not been previously reported and hence there is no clear explanation for the greater effects of diuron on roots than shoots. However, recent research investigated the phytotoxic effects of diuron on a leguminous crop belonging to the Fabaceae family, sown in an acidic sandy loam soil in Northam, Western Australia, revealing significant decreases in nodulation, nitrogen-fixing ability, and peak biomass of the crop [60]. To better understand the effects of diuron on the root system of chickpea, further study is required to verify whether the herbicide may affect nodule formation and limit N_2_ fixation as herbicides can inhibit the rhizobial–legume symbiosis in different ways, including direct effects on host plants, reducing nodule formation by inhibiting growth and survival of rhizobia, affecting the ability of the rhizobia to nodulate and retarding the enzymes or biochemical pathways for N_2_ fixation [61].

At the recommended label rate (405 g a.i. ha^-1^, equivalent to 0.25–0.29 mg kg^-1^) for sand and loamy sand, diuron caused approximately 60 and 40% root and shoot length inhibition of wheat in comparison to an untreated control. In our study, the ED_50_ for shoot length response in both soil types (2.0 mg kg^-1^) was observed at the highest application dose and it is similar to the previous finding by El-Nahhal and Hamdona [62], who reported that diuron has an EC_50_ of 1.83 mg kg^-1^ soil for shoot length reduction of wheat.

### Effect of soil properties on diuron phytotoxicity based on dose–response curve

The effect of diuron on shoot and root length and biomass inhibition of investigated species varied between the tested soils (Figs 2 and 3). Approximately 2–4 times and 2 times as much herbicide was required to produce 20 and 50% inhibition in shoot dry biomass of canola and wheat species on loamy sand as on sand. ED_50_ values for root dry weight of species were also 1.5–6 times higher in loamy sand compared to sand. While the ED_50_ for root length response of wheat and chickpea was similar between two soil types, canola had 2.7 times higher ED_50_ value in loamy sand than sand. This is presumably due to a lower bioavailability of herbicide in loamy sand as soil organic matter content is much higher than sand (Table 1) [63, 64]. The adsorption of diuron to organic matter and clay, mainly through hydrogen bonding, significantly decreases the herbicide’s bioavailability [65, 66]. Sorption of diuron onto the soil is known to increase in soils that have high organic matter content [67–70] which can reduce herbicide bioavailability and therefore toxicity [71–73]. In addition, the cation exchange capacity has a positive effect on diuron adsorption to the soil [64, 74] which is consistent with the lower phytotoxic effect in loamy sand that had a nine-fold higher cation exchange capacity (CEC) than sand (Table 1). Guimarães, Mendes [75] also found in loamy sand with high CEC (148.3 mmol dm^-3^) that there was a short half-life of diuron, due to increased nutrient availability and higher microbial activity which can increase diuron degradation. They concluded that organic carbon content and CEC of soils may affect both of the mineralization and degradation rate of diuron. Because soil factors such as organic matter and cation exchange capacity strongly influence the bioavailability and phytotoxic effects of diuron on plants, we recommend that a greater diversity of the soil types with varying CEC and organic matter content should be further tested to determine the diuron threshold levels in different soils before growing rotational crops.

### Implications for soil ED values

According to our study, the ED_50_ for shoot length in wheat occurred at the maximum tested rate but the ED_20_ occurred at rates lower than the recommended application rate. ED_20_ values representing a biomass reduction of 20% would also be considered important to farmers for identifying the toxicity threshold levels of diuron herbicide that depress crop growth causing potential economic losses. According to the soil survey report in 2015 [30] 35% of 40 broadacre cropping fields around Australia contained measurable diuron residues and the estimated residue levels in the WA field soil were 170 to 290 g a.i. ha^-1^ (0.12 to 0.2 mg kg^-1^). In 2016, over 30% of field soil samples from 84 Australian surveyed sites contained 0.012 to 0.275 mg kg^-1^ of diuron residues [4]. The maximum ED_20_ values for biomass reduction from our dose−response results for sand and loamy sand are approximately 0.02 and 0.09 mg kg^-1^, respectively. The maximum ED_50_ values for dry biomass reduction by diuron corresponded in chickpea with 0.48 and 0.24 mg kg^-1^ for sand and loamy sand, respectively. As chickpea is relatively tolerant to diuron, their response to diuron were not much different between soil types, indeed there was a higher ED_50_ in sand than in loamy sand. These values are relatively lower than field soil estimated residue levels and the label rate of 0.25 and 0.29 mg kg^-1^. Toxicity thresholds for ED_20_ and ED_50_ that are lower than the levels of diuron residue in agricultural soils in WA is a matter of concern particularly for sand and loamy sand soils. Hence, further research should be conducted to determine which factors influence the bioavailability of diuron in soils, the breakdown and degradation of diuron, and how they affect the ED values of various soil types.

## Conclusion

The plant bioassay using dose responses determined phytotoxicity thresholds for diuron residues present in sand and loamy sand soils. Among the different approaches tested, the four and three-parameter log-logistic model provided the best fit for assessing the soil herbicide threshold levels based on the response of crops to diuron. Canola was the most sensitive to diuron followed by wheat and chickpea, and crops were more sensitive in the low organic matter sand compared with the loamy sand, which contained higher organic matter. Except for chickpea, the diuron concentrations causing 50% biomass reduction in canola and wheat grown in sand and loamy sand (0.14 and 0.24 mg kg^-1^) were found to be below the label rates (0.25 and 0.29 mg kg^-1^) and also below the average and maximum diuron residue loads (0.17 and 0.29 mg kg^-1^) estimated in recent surveys on Western Australian crop fields. The ED_50_ for wheat shoot length occurred at the highest tested rate, whereas the ED_20_ was observed below the recommended application rate. Farmers would also find the ED_20_ values for biomass reduction crucial, as they indicate the toxicity threshold levels of diuron that can reduce crop growth and potentially lead to economic losses. Further research is needed to determine the effect of organic matter, clay levels, pH, and cation exchange capacity on the phytotoxicity thresholds of diuron to non-target crops, as these soil properties can influence the bioavailability, persistence, and overall toxicity of diuron, thereby affecting crop health and productivity.

## Supporting information

S1 Table Mixture of nutrient solution. (DOCX)

S2 Table Dose−response models used for model selection. (DOCX)

S3 Table Mean plant responses (%) corresponding to diuron doses for loamy sand and sand with ± SE (n = 3). (DOCX)

S4 Table Results of the model selection for chickpea root biomass response to diuron. (DOCX)

S5 Table Lack-of-fit test. (DOCX)

S6 Table Comparison of fitting log-logistic models. (DOCX)

S1 Data (XLSX)
