# Supplementary material for: Phytotoxicity risk assessment of diuron residues in sands on wheat, chickpea, and canola
Source: PLoS One. 2024 Dec 6;19(12):e0306865. doi: 10.1371/journal.pone.0306865 (PMC11623473; doi:10.1371/journal.pone.0306865)
Supplement: S1 Table — (DOCX) [file pone.0306865.s001.docx]

**Supporting information**

| **S1 table. Mixture of nutrient solution.** | | | | | |
| --- | --- | --- | --- | --- | --- |
| **Chemical** | **MW** | **uM** | **g/L for 1000Xstock solution** | **Wt of chemical (g)** | **Volume of nutrient solution** |
| NH4NO3 | 80.04 | 4000 | 160.08 | 8.004 | 50 ml solution |
| KH2PO4 | 136.09 | 500 | 68.045 | 3.40225 | 50 ml solution |
| CaCl2.2H2O | 147.02 | 1000 | 147.02 | 7.351 | 50 ml solution |
| MgSO4.7H2O | 246.77 | 500 | 123.385 | 6.16925 | 50 ml solution |
| MnSO4.H2O | 169.01 | 2 | 0.33802 | 0.33802 | 1000 ml |
| ZnSO4.7H2O | 287.54 | 2 | 0.57508 | 0.57508 | 1000 ml |
| CuSO4.5H2O | 249.68 | 0.5 | 0.12484 | 0.12484 | 1000 ml |
| H3BO3 | 61.83 | 9.8 | 0.605934 | 0.605934 | 1000 ml |
| Na2MoO4.2H2O | 241.95 | 0.08 | 0.019356 | 0.019356 | 1000 ml |
| *Note.* Stock solution is 1000X concentration so for watering plants, 10ml of each stock solution add to a container and make it up to 10L with DI water. | | | | | |
