# Supplementary material for: Phytotoxicity risk assessment of diuron residues in sands on wheat, chickpea, and canola
Source: PLoS One. 2024 Dec 6;19(12):e0306865. doi: 10.1371/journal.pone.0306865 (PMC11623473; doi:10.1371/journal.pone.0306865)
Supplement: S2 Table — (DOCX) [file pone.0306865.s002.docx]

**Supporting information**

| **S2 table. Dose response models used for model selection.** | |
| --- | --- |
| **Model name** | **Equation** |
| Four parameter log-logistic model | $Y=C+\frac{D-C}{1+\exp\left( B \left( \log\left( X \right)-\log\left( E \right) \right) \right)}$ |
| Three parameter log-logistic model | $Y=\frac{D}{1+\exp\left( B \left( \log\left( X \right)-\log\left( E \right) \right) \right)}$ |
| Four parameter Weibull type1 model | $Y=C+\left( D-C \right)\exp\left( -\exp\left( B\left( \log\left( X \right)-\log\left( E \right) \right) \right) \right)$ |
| Four parameter Weibull type 2 model | $Y=C+\left( D-C \right)\left( 1-\exp\left( -\exp\left( B \left( \log\left( X \right)-\log\left( E \right) \right) \right) \right) \right)$ |
| Y is the response of plant growth variable and C denotes the lower limit of the response when the dose X approaches infinity; D is the upper limit when the dose X approaches 0. B is the slope around the point of inflation (ED_50_), which identified the dose that causes a 50% response of length or biomass between the upper and lower limit. | |
