# Supplementary material for: Phytotoxicity risk assessment of diuron residues in sands on wheat, chickpea, and canola
Source: PLoS One. 2024 Dec 6;19(12):e0306865. doi: 10.1371/journal.pone.0306865 (PMC11623473; doi:10.1371/journal.pone.0306865)
Supplement: S3 Table — (DOCX) [file pone.0306865.s003.docx]

**Supporting information**

| **S3 table. Mean plant responses (%) corresponding to diuron doses for loamy sand and sand with ± SE (n=3).** | | | | | | | | | | | | |
| --- | --- | --- | --- | --- | --- | --- | --- | --- | --- | --- | --- | --- |
| Plant responses (%) | | | | | | | | | | | | |
| Doses (mg/kg) | SDWI | RDWI | SLI | RLI | SDWI | RDWI | SLI | RLI | SDWI | RDWI | SLI | RLI |
| Loamy sand | Canola | | | | Chickpea | | | | Wheat | | | |
| 0 | 0 ± 0^b^ | 0 ±0^b^ | 0 ±0^c^ | 0 ±0^b^ | 0 ±0^d^ | 0 ±0^c^ | 0 ±0^c^ | 0 ±0^c^ | 0 ±0^d^ | 0 ±0^cd^ | 0 ±0^d^ | 0 ±0^d^ |
| 0.03 | 13±21^b^ | -13±11^b^ | 1 ±1^c^ | -2 ±14^b^ | 19 ±7^cd^ | 28 ±5^b^ | 1 ±2^c^ | 16 ±5^bc^ | -0.1±0.3^d^ | -13 ±4^d^ | 0.1 ±1^d^ | 13 ±0.1^cd^ |
| 0.05 | 30±10^b^ | 25±22^b^ | 5 ±3^c^ | 15±10^b^ | 18±7^cd^ | 33 ±7^b^ | 2 ±2^c^ | 21 ±8^bc^ | 7 ±2^d^ | 13±8^bc^ | 6 ±1^d^ | 14 ±2^c^ |
| 0.1 | 81±2^a^ | 89±0^a^ | 20 ±2^b^ | 69 ±2^a^ | 28 ±1^c^ | 41 ±3^b^ | 3 ±1^c^ | 24 ±1^b^ | 25 ±3^c^ | 35 ±6^b^ | 11 ±2^d^ | 29 ±4^b^ |
| 0.29 | 100±0^a^ | 100±0^a^ | 100±0^a^ | 100±0^a^ | 61±2^b^ | 71±3^a^ | 16±3^b^ | 54±6^a^ | 53±6^b^ | 64±1^a^ | 36 ±2^c^ | 64 ±4^a^ |
| 0.87 | 100±0^a^ | 100±0^a^ | 100±0^a^ | 100±0^a^ | 74 ±5^ab^ | 77 ±1^a^ | 34±2^a^ | 58 ±1^a^ | 89 ±0.8^a^ | 80±3^a^ | 41 ±1^b^ | 74 ±0.7^a^ |
| 1.74 | 100±0^a^ | 100±0^a^ | 100±0^a^ | 100±0^a^ | 79 ±4^ab^ | 80 ±4^a^ | 36±4^a^ | 62 ±4^a^ | 93 ±3^a^ | 81±3^a^ | 44 ±0.3^a^ | 73 ±4^a^ |
| 2.61 | 100±0^a^ | 100±0^a^ | 100±0^a^ | 100±0^a^ | 82 ±2^a^ | 79 ±3^a^ | 39±2^a^ | 64 ±5^a^ | 91±2^a^ | 83±7^a^ | 46±0.2^ab^ | 75 ±3^a^ |
| Sand | Canola | | | | Chickpea | | | | Wheat | | | |
| 0 | 0 ±0^e^ | 0 ±0^c^ | 0±0^d^ | 0 ±0^c^ | 0 ±0^c^ | 0 ±0^c^ | 0 ±0^b^ | 0 ±0^d^ | 0 ±0^e^ | 0 ±0^d^ | 0 ±0^d^ | 0 ±0^d^ |
| 0.03 | 57±2^d^ | 67±10^b^ | 20±3^c^ | 54 ±4^b^ | 4 ±13^c^ | 24 ±9^b^ | 5±8^ab^ | 18±10^cd^ | 24 ±2^d^ | 39 ±1^c^ | 10±1^cd^ | 18±10^cd^ |
| 0.04 | 67±3^c^ | 78±6^b^ | 28±2^c^ | 62 ±5^b^ | 23±13^bc^ | 43 ±9^b^ | 7 ±6^ab^ | 37±6^bc^ | 42 ±4^c^ | 39 ±1^c^ | 20±4^bc^ | 37 ±6^bc^ |
| 0.08 | 83±1^b^ | 78±6^b^ | 52±6^b^ | 61 ±3^b^ | 44±5^ab^ | 78 ±1^a^ | 16±4^ab^ | 40±6^bc^ | 56±7^bc^ | 47 ±4^bc^ | 31 ±9^ab^ | 40 ±6^bc^ |
| 0.25 | 100±0^a^ | 100±0^a^ | 100±0^a^ | 100±0^a^ | 49±1^ab^ | 77 ±1^a^ | 23±2^a^ | 53 ±3^ab^ | 63±2^ab^ | 54 ±3^b^ | 40 ±3^a^ | 53 ±3^ab^ |
| 0.75 | 100±0^a^ | 100±0^a^ | 100±0^a^ | 100±0^a^ | 57±1^a^ | 78 ±0^a^ | 22±4^ab^ | 65 ±1^a^ | 67±3^ab^ | 67 ±1^a^ | 41 ±2^a^ | 65 ±1^a^ |
| 1.5 | 100±0^a^ | 100±0^a^ | 100±0^a^ | 100±0^a^ | 56±2^ab^ | 76 ±1^a^ | 17±5^ab^ | 67 ±3^a^ | 72±1^a^ | 66 ±3^a^ | 46 ±2^a^ | 67 ±3^a^ |
| 2.25 | 100±0^a^ | 100±0^a^ | 100±0^a^ | 100±0^a^ | 62±4^a^ | 81 ±0^a^ | 26±4^a^ | 66 ±1^a^ | 76±1^a^ | 70 ±2^a^ | 47 ±2^a^ | 66 ±1^a^ |

*Note-* Significant differences between different doses shown as different letters from the Tukey’s HSD PostHoc test.

SDWI*,* Shoot dry weight inhibition; RDWI, Root dry weight inhibition; SLI, Shoot length inhibition; RLI, Root length inhibition.

Within a column, means with same letters are not significantly different (P < 0.05, Tukey’s HSD test).
