# Supplementary material for: Phytotoxicity risk assessment of diuron residues in sands on wheat, chickpea, and canola
Source: PLoS One. 2024 Dec 6;19(12):e0306865. doi: 10.1371/journal.pone.0306865 (PMC11623473; doi:10.1371/journal.pone.0306865)
Supplement: S4 Table — (DOCX) [file pone.0306865.s004.docx]

**Supporting information**

| **S4 table. Results of the model selection for chickpea root biomass response to diuron.** | | | |
| --- | --- | --- | --- |
| **Models** | **AIC** | **Lack of fit** | **Res var** |
| LL.4 | -154.8 | 0.85 | 7.3e^-05^ |
| W2.4 | -153.6 | 0.68 | 7.7e^-05^ |
| W2.3 | -126.5 | 2.5e^-04^ | 2.5e^-04^ |
| LL.3 | -124.6 | 2.7e^-04^ | 2.7e^-04^ |
| *Note.* LL.4 is four-parameter log-logistic model, W2.4 is four-parameter Weibull type 2 model, W2.3 is three-parameter Weibull type 2 model, LL.3 is three-parameter log-logistic model, AIC is Akaike's information criteria, Lack of fit is representing for whether a regression model is a poor model of the data, Res var is residual variance. | | | |
