# Supplementary material for: Phytotoxicity risk assessment of diuron residues in sands on wheat, chickpea, and canola
Source: PLoS One. 2024 Dec 6;19(12):e0306865. doi: 10.1371/journal.pone.0306865 (PMC11623473; doi:10.1371/journal.pone.0306865)
Supplement: S5 Table — (DOCX) [file pone.0306865.s005.docx]

**Supporting information**

| **S5 table. Lack-of-fit test.** | | | | | |
| --- | --- | --- | --- | --- | --- |
| **Model** | **Df** | **RSS** | **Df** | **F value** | **p value** |
| ANOVA | 16 | 3241.7 |  |  |  |
| LL.4 | 20 | 0.001 | 4 | 0.34 | 0.85 |
| W2.4 | 20 | 0.002 | 4 | 0.58 | 0.68 |
| W2.3 | 21 | 0.005 | 5 | 9.1 | 0.0003 |
| LL.3 | 21 | 0.006 | 5 | 10.12 | 0.0002 |
