# Supplementary material for: Phytotoxicity risk assessment of diuron residues in sands on wheat, chickpea, and canola
Source: PLoS One. 2024 Dec 6;19(12):e0306865. doi: 10.1371/journal.pone.0306865 (PMC11623473; doi:10.1371/journal.pone.0306865)
Supplement: S6 Table — (DOCX) [file pone.0306865.s006.docx]

**Supporting information**

| **S6 table. Comparison of fitting log-logistic models.** | | | |
| --- | --- | --- | --- |
| **Crop and Soil type** | **Plant growth parameters** | **Fitting models** | |
| Canola-Sand | SDW | LL.4 | LL.3 |
|  | RDW | LL.4 | LL.3 |
|  | SL | LL.4 | LL.3 |
|  | RL | LL.4 | LL.3 |
| Chickpea-Sand | SDW | LL.4 | LL.3 |
|  | RDW | LL.4 |  |
|  | SL | LL.4 | LL.3 |
|  | RL | LL.4 | LL.3 |
| Wheat-Sand | SDW | LL.4 | LL.3 |
|  | RDW | LL.4 | LL.3 |
|  | SL | LL.4 |  |
|  | RL | LL.4 | LL.3 |
| Canola-Loamy sand | SDW | LL.4 | LL.3 |
|  | RDW | LL.4 | LL.3 |
|  | SL | LL.4 | LL.3 |
|  | RL | LL.4 | LL.3 |
| Chickpea-Loamy sand | SDW | LL.4 | LL.3 |
|  | RDW | LL.4 | LL.3 |
|  | SL | LL.4 |  |
|  | RL | LL.4 | LL.3 |
| Wheat-Loamy sand | SDW | LL.4 | LL.3 |
|  | RDW | LL.4 | LL.3 |
|  | SL | LL.4 |  |
|  | RL | LL.4 | LL.3 |
